# Recent Perspective of *Lactobacillus* in Reducing Oxidative Stress to Prevent Disease

**DOI:** 10.3390/antiox12030769

**Published:** 2023-03-21

**Authors:** Tingting Zhao, Haoran Wang, Zhenjiang Liu, Yang Liu, DeJi, Bin Li, Xiaodan Huang

**Affiliations:** 1 School of Public Health, Lanzhou University, Lanzhou 730033, China; 2 Institute of Animal Husbandry and Veterinary, Tibet Academy of Agricultural and Animal Husbandry Sciences, Lhasa 850000, China; 3 National Engineering Laboratory for AIDS Vaccine, School of Life Sciences, Jilin University, Changchun 130012, China

**Keywords:** *Lactobacillus*, oxidant stress, IBD, cancer, liver diseases, MAPK, NF-κB, Nrf2

## Abstract

During oxidative stress, an important factor in the development of many diseases, cellular oxidative and antioxidant activities are imbalanced due to various internal and external factors such as inflammation or diet. The administration of probiotic *Lactobacillus* strains has been shown to confer a range of antibacterial, anti-inflammatory, antioxidant, and immunomodulatory effects in the host. This review focuses on the potential role of oxidative stress in inflammatory bowel diseases (IBD), cancer, and liver-related diseases in the context of preventive and therapeutic effects associated with *Lactobacillus*. This article reviews studies in cell lines and animal models as well as some clinical population reports that suggest that *Lactobacillus* could alleviate basic symptoms and related abnormal indicators of IBD, cancers, and liver damage, and covers evidence supporting a role for the Nrf2, NF-κB, and MAPK signaling pathways in the effects of *Lactobacillus* in alleviating inflammation, oxidative stress, aberrant cell proliferation, and apoptosis. This review also discusses the unmet needs and future directions in probiotic *Lactobacillus* research including more extensive mechanistic analyses and more clinical trials for *Lactobacillus*-based treatments.

## 1. Introduction

### 1.1. Oxidative Stress and Disease

Oxidative stress is associated with the accumulation of excess reactive oxygen and reactive nitrogen species (ROS and RNS, respectively, see Table S1). In cells, ROS/RNS are continuously produced through metabolic processes. Low to moderate ROS levels serve as essential secondary messengers in cell signaling, regulating cell proliferation, differentiation, and migration, and participate in triggering cellular survival mechanisms. However, excessive ROS/RNS can cause oxidative damage to a variety of biomolecules such as unsaturated fatty acids, proteins, and DNA [1]. Endogenous ROS are principally produced in mitochondria, the endoplasmic reticulum, and peroxisomes, with the vast majority of ROS generated by the mitochondrial electron transport chain [2,3]. In addition, numerous enzymes are known to participate in catalyzing the production of endogenous ROS/RNS such as peroxidases, nicotinamide adenine dinucleotide phosphate (NADPH) oxidase (NOX), myeloperoxidase (MPO), nitric oxide synthase (NOS), lipoxygenases (LOXs), and cyclooxygenases (COXs) [4,5]. Beyond these enzymatic pathways for ROS/RNS production, exposure to a wide range of exogenous factors such as trans-fatty acids, iron (Fe), and copper (Cu) in foods, ultraviolet radiation, various drugs and xenobiotics as well as chronic infection and inflammatory diseases can also contribute to inducing ROS accumulation [5,6,7,8].

In humans and other mammals, antioxidant defense systems can largely counteract the effects of ROS/RNS. Endogenous antioxidant systems are generally classified as belonging to either the intracellular enzymatic antioxidants, comprising superoxide dismutase (SOD), catalase (CAT), and other such antioxidant enzymes, or the intracellular non-enzymatic antioxidants, which rely on the activity of small molecules in conjunction with enzymes to neutralize ROS/RON. In the latter system, glutathione functions as a significant antioxidant barrier in the gut, along with three related enzymes: glutathione peroxidase (GPX), glutathione reductase (GSR), and glutathione S-transferase (GST) [1,9]. In addition, the melatonin (MEL) and thioredoxin (Trx) system are well-studied, essential, intracellular antioxidants [10,11]. In addition to the above, there are also some extracellular antioxidants such as vitamin A/C or *β*-carotene obtained from vegetables and fruits, or flavonoids from certain plants [12,13]. Oxidative stress can occur when ROS/RNS generation exceeds the cellular capacity for scavenging activity of the antioxidant systems or as a result of the dysregulation of antioxidant pathways [5], leading to the damage of various physiological systems in the body. Some examples of the pathological damage related to ROS/RNS accumulation include atherosclerosis in the cardiovascular system; neurodegenerative diseases of the nervous system such as Alzheimer’s disease and Parkinson’s disease, rheumatoid arthritis or systemic lupus erythematosus of the autoimmune system, as well as IBD, stomach cancer, colorectal cancer, and other diseases of the digestive system [14,15].

Alleviating oxidative stress is thus essential for treating many diseases and ensuring systemic health. As our understanding of ROS/RNS and antioxidant mechanisms expands, more precise therapeutic interventions can be developed that focus on disease-related sources and targets of ROS/RNS, some of which are currently entering the clinical trial stages of testing [16]. For instance, free iron is a promising target to control the site and extent of highly aggressive hydroxyl radical generation, which could benefit the treatment of oxidative stress-related diseases [8]. There is also potential for the application of more targeted ROS/RNS inhibitors such as site-specific suppressors of superoxide production (i.e., S1QELs) in quinone-mediated reactions [14,17]. In addition, probiotics such as *Lactobacillus* and *Bifidobacterium* may serve as effective complementary therapies that enhance antioxidant defenses via ROS/RNS removal, the inhibition of pro-oxidative enzymes, and the synthesis of antioxidant enzymes. 

### 1.2. Antioxidant Activity of Lactic Acid Bacteria 

*Lactobacillus*, a genus comprising Gram-positive, aerotolerant, rod-shaped, non-sporulating species, is commonly found among microbiota in the gastrointestinal tract and is also frequently isolated from many fermented food products. This genus currently includes 253 published, validated species [18], and accounts for an estimated 6.0% of total bacterial cell numbers in the human duodenum and ~0.3% of all bacteria in the colon [19]. The beneficial and functional properties of *Lactobacillus* in promoting human health have been extensively documented in recent years. Members of *Lactobacillus* have been shown to adhere to the intestinal epithelium and produce antimicrobial metabolites such as ethanol, hydrogen peroxide (H_2_O_2_), acetic acid, lactic acid, and/or succinic acid, which can antagonize other, potentially pathogenic, bacteria [20]. Some *Lactobacillus* strains can reportedly enhance cellular immune responses via the activation of macrophages, natural killer (NK) cells, and/or antigen-specific cytotoxic T-lymphocytes, or by stimulating the release of various cytokines. Moreover, some *Lactobacillus* species could also improve the immune response in gut mucosa by promoting the recruitment of IgA(+) cells [21]. Some *Lactobacillus* species may also exhibit direct antioxidant activity, and a few species are known to possess oxidative stress resistance genes and proteins pivotal for redox mechanisms such as *katA* expressed by *L. sakei*, or thioredoxin antioxidant system proteins expressed by some *L. plantarum* and *L. casei* strains [18]. Apart from these effects, *Lactobacillus* species also produce functional products such as exopolysaccharides (EPS), which participate in mitigating oxidative damage or activating the expression of host transcription factors involved in modulating cellular oxidative stress [22,23].

In recent years, several studies have investigated the relationship between *Lactobacillus* and oxidative stress in the host. Due to the purported beneficial role of *Lactobacillus* in human health, research has increasingly focused on the positive impacts of these species in ameliorating oxidative stress and related diseases [18]. Moreover, further in-depth exploration of the role of oxidative stress in disease will provide new perspectives that contribute to improving clinical treatments. This review covers the pathological mechanisms of oxidative stress in inflammatory bowel disease, cancer, and liver-related diseases as well as the preventive and therapeutic effects of *Lactobacillus* in patients with these diseases.

## 2. Inflammatory Bowel Disease

### 2.1. Inflammatory Bowel Disease and Oxidative Stress

Inflammatory bowel disease (IBD) including Crohn’s disease (CD) and ulcerative colitis (UC) is characterized by chronic, non-specific intestinal inflammation. CD can affect the entire alimentary tract from the oral cavity to the anus (mainly the terminal ileum and adjacent colon) and include lesions that appear in a segmental or stochastic (i.e., not continuous) distribution and commonly lead to complications such as abscesses, fistula, and stenosis. In contrast, these lesions are absent in UC, which is characterized by mucosal inflammation and is limited to the colon [24]. Previous studies have shown that individual genetic susceptibility, external environment, gut microbiota, immune response, and oxidative stress are all contributing factors that can be firmly linked to the pathogenesis of IBD [25,26], with lipid peroxidation (LPO) and ROS accumulation, in particular, serving as important causes of oxidative stress in the intestine. 

Several points have been proposed regarding the relationship between oxidative stress and IBD. First, researchers have identified several genetic risk loci associated with IBD, and mutations in genes encoding antioxidant/biotransformation enzymes can negatively impact their activity and increase the risk of IBD. For example, case-control studies found that inter-individual polymorphisms related to a C609T conversion in NAD(P)H: quinone oxidoreductase 1 (NQO1), an enzyme involved in inflammation and oxidative stress response, might play a significant role in the development of colon cancer and could influence steroid resistance in UC patients [27,28]. In addition, a polymorphism in the promoter region of the Nuclear factor E2-related factor 2 (Nrf2) gene, which participates in regulating the expression of detoxifying and antioxidant proteins in the intestine, has been associated with UC development in a Japanese population [29]. Second, mucosal immune cells and intestinal epithelial cells (IECs) are involved in oxidative stress in the intestine and have been shown to play a central role in the pathogenesis of IBD. During mucosal inflammation, IECs as well as neutrophils and macrophages, produce superoxide and nitric oxide by activating NOX and inducible nitric oxide synthase (iNOS), respectively, both induced by inflammatory cytokines, eventually leading to the production of more ROS/RNS. The ROS/RNS overload alters the structure and function of the intestinal epithelium, damages cytoskeletal proteins, and accelerates cellular damage through lipid peroxidation, ultimately resulting in barrier destruction [5,30,31]. Third, mutual interactions between gut microbiota and oxidative stress have been identified in IBD. Notably, the composition of gut microbial taxa is altered in CD patients compared to that in healthy controls, characterized by increased relative abundance of *Bacteroidetes* and decreased *Firmicutes*. In addition, *Enterobacteriaceae*, especially *E. coli*, are enriched in CD patients [32]. ROS/RNS are associated with intestinal dysbiosis. During inflammation, gut microbiota can directly generate ROS/RNS, or indirectly induce the production of excessive ROS/RNS by activating neutrophils or gastrointestinal epithelial cells, stimulating an initial inflammatory response via positive feedback, which leads to further ROS/RNS production, aggravating intestinal oxidative stress and damaging tissues [5,33]. Therefore, restoring the normal structure of gut microbiota has positive implications for the alleviation of inflammation and oxidative stress in IBD. Lifestyle-associated factors can also contribute to gastrointestinal oxidative stress. Although the underlying mechanisms remain unclear, many studies have shown that nicotine intake from smoking can play a dual role in CD and UC, and smoking is a risk factor for the occurrence of CD, but has apparently protective effects in UC, according to epidemiological data [34,35]. Similarly, wine consumption may have various effects in IBD patients. Some chemicals such as polyphenols in red wine appear to confer antioxidant properties [36], whereas the alcohol itself can lead to inflammation and oxidative stress in the liver and intestines, and chronic alcohol consumption leads to increased risk of gastric or colon cancer [37].

**Figure 1:**
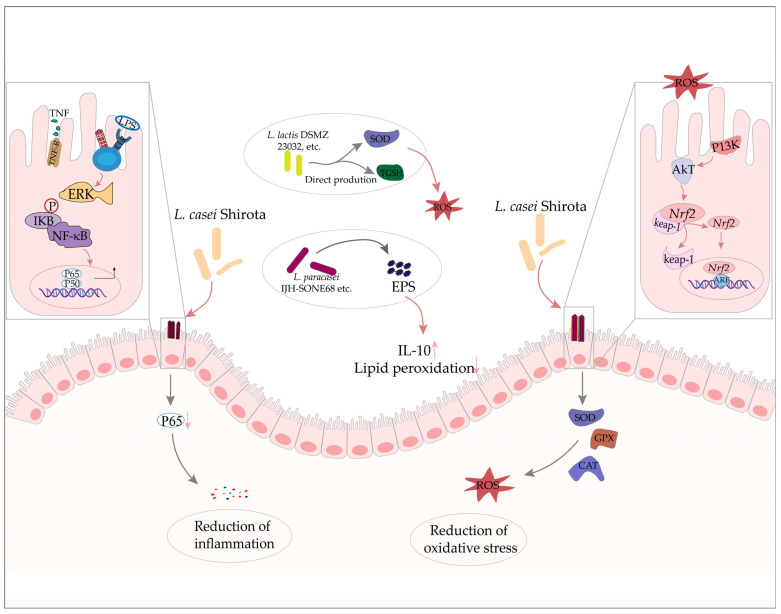
The role of *Lactobacillus* in IBD. *Lactobacillus* can produce EPS or/and related antioxidant enzymes to alleviate intestinal inflammation and oxidative stress, and ameliorate IBD by regulating the NF-κB, Nrf2 signaling pathway.

At present, treatments for IBD are still based on conventional anti-inflammatory agents (e.g., sulfasalazine and mesalazine, etc.) and immune modulators (e.g., thiopurines and cyclosporine, etc.). However, these drugs have severe side effects [38]. The incidence of UC and CD are higher in regions of Asia with high population density and coastal areas of China also have high rates of IBD, indicating that newer, more effective therapeutic interventions for IBD are urgently needed [39]. The beneficial effects of *Lactobacillus* in IBD have stimulated considerable research attention that has led to increased the clinical application of probiotic *Lactobacillus* strains as alternative or complementary therapies [40] (Figure 1). 

### 2.2. The Effects Lactobacillus in Inflammatory Bowel Disease 

A recent study exploring the effects of EPS-producing probiotic bacteria found that antioxidant enzyme activities were higher and lipid peroxidation was significantly ameliorated in UC model rats treated with probiotics compared with those in untreated UC model rats. More importantly, improvements to oxidative stress were significantly greater in rats administered with a high EPS producing strain than in those treated with a low EPS strain [41]. Similarly, another study showed that administering a novel EPS produced by *L. paracasei* IJH-SONE68 led to reduced macrophage inflammatory protein 2 mRNA levels, and the increased expression of the anti-inflammatory cytokine, interleukin-10 (IL-10) in dextran sulfate sodium (DSS)-induced UC model mice [42] (Figure 1). These results suggest that EPS should be further examined for application in new treatments for IBD in the future, and *Lactobacillus* would be among one of the best choices for EPS production.

In the IBD model mice, administration of *L. plantarum* ZS62 was found to inhibit DSS-induced colonic atrophy. Moreover, mice supplemented with strain ZS62 had lower serum levels of oxidative stress indicators (i.e., Malondialdehyde and MPO) and inflammatory indicators (i.e., IL-1β, IL-6, IL-12, TNF-α, etc.) at both the mRNA and protein levels and elevated protein and mRNA levels of antioxidant enzymes (i.e., CAT, T-SOD) and IL-10 [43]. In addition, Chorawala and colleagues reported that exposure to the cell wall components of *L. casei*, *L. acidophilus*, and *L. rhamnosus* could attenuate lipopolysaccharide (LPS)-induced rats colitis by modulating the inflammatory immune response and oxidative stress [44]. Studies have suggested that *Lactobacillus* can improve the hosts’ antioxidant level. For example, Erdogan et al. found that feeding fermented Kefir significantly increased the serum total antioxidant status (TAS) levels in Balb/c mice [45]. Similarly, in a healthy volunteer trial (Application No. WO03002131), study subjects who consumed *L. fermentum* ME-3 in fermented goat milk or who received capsules with *L. fermentum* ME-3 displayed significantly higher blood total antioxidative activity and TAS than subjects who did not [46]. Furthermore, a pediatric, randomized, placebo-controlled trial demonstrated that a highly concentrated mixture of probiotic bacterial strains (VSL#3) was both safe and effective in maintaining remission among patients with active UC [47]. However, another trial in which CD patients were administered 1 × 10^9^ CFUs of the probiotic strain *L. johnsonii* LA1 four times daily showed no preventive effects on endoscopic disease recurrence [48]. 

Assessment of the antioxidant capacity of 34 lactic acid bacteria strains in vitro by Amaretti et al. indicated that strains such as *L. lactis* DSMZ 23032, *L. acidophilus* DSMZ 23033, and *L. brevis* DSMZ 23034 produced relatively high intracellular levels of glutathione and SOD [49], suggesting that LAB might be effective for delivering these antioxidant enzymes to the gut to alleviate oxidative stress in IBD (Figure 1). Furthermore, the co-expression of antioxidant enzyme genes in *Lactobacillus* through genetic engineering could enhance their antioxidant capacity while also increasing their viability in the host by several-fold, supporting their potential development in therapeutics for IBD [50,51,52]. 

Finamore et al. showed that pretreatment of the enterocyte-like cell line TC7/human colon carcinoma cell line (Caco-2) with *L. casei* Shirota (LcS) could prevent membrane barrier disruption and intercellular ROS accumulation, significantly increase the gastrointestinal expression of GPX, and reduce p65 phosphorylation. These findings support the involvement of theNfr2 and Nuclear Factor-Kappa B (NF-κB) pathways in the activation of antioxidant cellular defenses [53] (Figure 1). However, the induction of GPX has also been shown to occur in a Nrf2-independent manner via activation of the transcription factor, STAT3 [54]. In short, some *Lactobacillus* strains appear to confer positive effects in maintaining intestinal homeostasis via enhanced antioxidant capacity and inflammatory response, although the specific mechanisms through which *Lactobacillus* can alleviate oxidative stress in IBD remain unclear.

Although *Lactobacillus* is generally regarded as a safe microorganism, several studies have indicated that certain *species* may pose risks such as bacteremia and endocarditis [55,56]. Therefore, the application of *Lactobacillus* for the treatment of IBD raises concerns regarding the presence of virulence genes and antibiotic resistance genes, as their potential transfer to pathogenic microorganisms could constitute a risk [57].

## 3. Cancer

### 3.1. ROS and Cancer

Cancer is among the leading causes of death worldwide. According to the Global Cancer Statistics, approximately 19.3 million new cancer cases and nearly 10 million deaths from cancer were reported in 2020 [58]. Tumorigenesis may result from a combination of internal and external factors, some of which influence cancer progression and metastasis through increased ROS production [59]. The generation of ROS in cancer cells is primarily mediated by oxidative phosphorylation in the electron transport chain on the mitochondrial inner membrane. In addition, transition metals such as iron can also generate ROS non-enzymatically via a Fenton reaction, while exposure to various external factors such as air pollutants, radiation, foods, or drugs, etc. can also exogenously induce ROS production [60]. In general, the aberrant and uncontrolled proliferation of cancer cells requires large amounts of ATP as an energy supply, and the resulting metabolic upregulation leads to elevated accumulation of ROS. However, cancer cells have a characteristically high antioxidant capacity due to the Nrf2-mediated activation of various antioxidant response elements (AREs) that can restrict ROS accumulation to levels compatible with cellular biological functions, albeit still higher than that in normal cells [61].

At low to moderate levels, ROS may promote abnormal cell proliferation and differentiation by inducing DNA mutations These mutations mainly include DNA double strand breaks accompanied by abnormalities in their associated repair pathways, or by generating 8-oxo-7-hydrodeoxyguanosine (8-oxodG), which is recognized as an indicator of oxidative damage to DNA [62,63]. Other work has linked Fe-catalyzed ROS accumulation to the development of some cancers. For instance, a multivariate statistical analysis indicated that iron depletion therapy, either through phlebotomy or by maintaining a low-iron diet, could significantly lower the risk of hepatocellular carcinoma compared to that in untreated patients [64,65]. 

**Figure 2:**
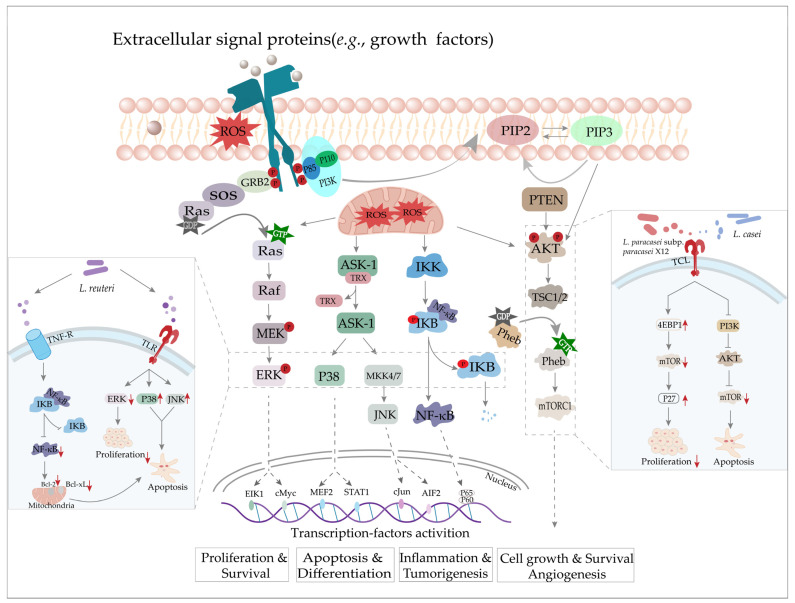
ROS induces the activation of the MAPK, NF-κB, and PI3K/PTEN signaling pathways to promote cancer development, progression, and apoptosis, etc. and aa possible target of LAB [59].

In addition, ROS may also contribute to tumorigenesis through their function as signal molecules (Figure 2). Increased ROS production in cells can induce an enzymatic cascade reaction that results in the activation of extracellular signal-regulated kinase (ERK), c-Jun N-terminal kinase (JNK), and p38 mitogen-activated protein kinases (MAPK), all of which are linked to tumor cell growth and survival [66,67]. Weinberg and colleagues observed that mitochondrial ROS are essential for Kras-mediated tumorigenesis in a murine model of lung carcinoma via ERK-MAPK signaling [59,68]. The phosphatidylinositol-3-kinase (PI3K)/phosphatase and tensin homolog deleted on chromosome 10 (PTEN) signaling pathway is also important for tumorigenesis and cancer metastasis. ROS can stimulate PI3K/protein kinase B (AKT) signaling through the oxidation of a cysteine thiol group on various phosphatases (e.g., PTEN, PTP1B, PP2A) and contribute to dysregulation in a wide range of human cancers (e.g., endometrial, breast, thyroid, and prostate cancers) [69,70]. In addition to the above pathways, NF-κB is increasingly recognized as a key player in many steps of cancer development and progression. Treatment of breast carcinoma cells with IL-1β, TNFα, or sodium arsenite generates H_2_O_2_ and O^2−^, which in turn activate NF-κB and enhance uncontrolled cellular growth [71,72]. Constitutive activation of Nrf2 has been observed in various human cancers such as those affecting the lungs, breasts, and skin [73,74]. In addition, deregulation of the Nrf2–Keap1 pathway has been reported in multiple cancer cells, leading to drug resistance, genomic instability, resistance to apoptosis, metastasis, and metabolic [75].

However, high levels of ROS have contrary effects on cancer, promoting cell death and severe cellular damage. Some chemotherapeutic drugs or cytotoxic agents such as anthracyclines, platinum-based drugs (e.g., cisplatin, carboplatin), and other alkylating drugs exert therapeutic effects by stimulating the high intracellular production of ROS [76,77]. In addition, excessive ROS levels are also positively correlated with apoptosis through the ASK-1/JNK/p38 signaling cascade induced via the H_2_O_2_-mediated oxidation of cysteine residues on TRX, which leads to the dissociation of ASK-1, the subsequent activation of JNK and p38 signaling, and ultimately apoptosis [59,78] (Figure 2).

Thus, ROS can serve as a “double-edged sword”, both causing and suppressing cancer [79], and current studies suggest that modulating the oxidative stress response might be an effective, new potential approach for cancer therapies. 

### 3.2. The Role of Lactobacillus in Cancer

Drug resistance is a serious problem in cancer chemotherapy, and thus effective alternative therapeutic options are desperately needed to improve treatment response in many cancer patients. To address this issue, an increasing number of studies has investigated the potential antitumor activity of *Lactobacillus* and provide evidence supporting its further exploration and development for use in candidate anti-tumor therapies [80,81].

In many cancers, abnormal cell proliferation is tightly linked to the suppression of apoptosis, and many drugs achieve therapeutic efficacy by promoting apoptosis in cancer cells. Notably, *Lactobacillus* has been shown to induce apoptosis [82,83]. For example, oral pretreatment with the probiotic strain, *L. acidophilus* NCFM, was found to stimulate apoptosis in CT-26 cell dorsal flank xenograft tumors or in segmental orthotopic colon cancer in mice [84]. Another study showed that the apoptosis levels increased in the LS513 colorectal cancer cells exposed to a combination of the *L. acidophilus* and *L. casei* strains in the presence of 5-fluorouracil (5-FU) in a dose-dependent manner. This effect was potentially due to the faster activation of caspase-3 and the downregulation of the p21 protein [85]. Similarly, Altonsy et al. found that the *L. rhamnosus* GG strain (LGG) could induce caspase-9 and caspase-3 activation, promoting apoptosis in the Caco-2 colon cancer cells [86]. Homogenates of strain LGG were also found to induce the apoptosis pathways in HGC-27 human gastric cancer cells via reduced expression of the anti-apoptotic protein, Bcl-2, and elevated expression of the pro-apoptotic protein, Bax [87]. Similarly, ubiquitination of IKB was suppressed in human chronic myeloid leukemia-derived cells (KBM-5 cells) treated with *L. reuteri* ATCC PTA 6475, resulting in the downregulation of the NF-κB-dependent gene products (e.g., Bcl-2, Bcl-xL), while pre-treatment with *L. reuteri* enhanced JNK and p38 phosphorylation, but suppressed ERK1/2 signaling in cells, increasing the apoptosis levels and inhibiting proliferation, respectively [88]. In addition, Hwang and colleagues confirmed that extracts of *L. casei* could induce apoptosis in gastric cancer cells by suppressing PI3K/AKT signaling [89] (Figure 2). Thus, numerous studies have shown that *Lactobacillus* strains can regulate apoptosis in cancer cells via the modulation of endogenous signaling pathways or exogenous cues.

*Lactobacillus* strains and their metabolites (i.e., components of the cell wall and cytoplasm) have been documented to inhibit cancer cell proliferation. For instance, Orlando and colleagues investigated the anti-proliferative effects of the cell wall and cytoplasmic fractions of strain LGG on the HGC-27 cells and DLD-1 human colonic adenocarcinoma cells. They found that both HGC-27 and DLD-1 cells were resistant to the bacterial cell wall fraction, whereas treatment with the cytoplasmic fraction resulted in apparently reduced proliferation [90]. 4E-Binding protein 1 (4EBP1) is a transcriptional inhibitor in the downstream of the mTOR signaling pathway. Treatment with *L. paracasei* subsp. *paracasei* X12 could induce cell cycle arrest at G1 in the HT-29 cells through the mTOR-4EBP1-p27 signaling pathway, and the mRNA expression of 4EBP1 was increased several folds after treatment by the X12 strain [91]. Hence, blocking hyperactivation of the PI3K/AKT/mTOR signaling pathway has emerged as a plausible target for cancer therapies due to its involvement in cell growth and proliferation (Figure 2).

Polyamines including putrescine, spermidine, and spermine, etc. can serve as nutrient substrates for cancer cell growth and may thus play an important role in cancer cell proliferation. In gastric cancer cells, probiotics such as *Lactobacillus* strains can potentially act as anti-neoplastic agents to inhibit proliferation by modulating polyamine contents or function [92]. *L. salivarius* strains FP25 and FP35, isolated from infant feces, can directly adhere to cancer cells, triggering the biosynthesis of short-chain fatty acids (SCFAs), especially butyric and propionic acids, resulting in the inhibition of colon cancer cell proliferation [93]. In addition, some studies have reported that EPS secreted by *Lactobacillus* might also exert anti-proliferative effects on cancer cells [94,95,96].

A growing body of evidence supports that *Lactobacillus* strains can also provide antioxidant functions to prevent oxidative stress-related tumor development. A recently identified probiotic strain, *L. salivarius* REN, isolated from fecal samples of centenarians by Zhang and colleagues, was found to inhibit 4NQO (4-nitroquioline 1-oxide)-induced oral cancer in rats by reducing oxidative DNA damage and downregulating COX-2 expression [97]. In addition, *L. reuteri* is capable of regulating intestinal metabolites to promote a growth-repressive effect on cancer and reduce the oxidation level in mice with colorectal cancer [98]. In rats with colon cancer induced by 1,2-dimethylhydrazine (DMH), treatment with *L. plantarum* AS1 led to significant improvements in lipid peroxidation and antioxidant activities in the colon and plasma, and showed promising antioxidant effects in vitro [99]. Choi et al. found that heat-killed (HK) cells and soluble polysaccharide components of *L. acidophilus* 606 exhibited potent antioxidative activity, and further reported that the soluble polysaccharide fraction could induce apoptosis in HT-29 cells in vitro, suggesting that soluble polysaccharides of *Lactobacillus* strains could be explored as possible candidate anticancer agents [100]. Overall, many in vivo and in vitro studies support that *Lactobacillus* can reduce oxidative damage, attenuating DNA mutation and cancer development.

*Lactobacillus* can also exert immunomodulatory and cytotoxic anticancer effects. For instance, *L. casei* strains 9018 and LcS could activate NK cells by inducing the production of cytokines such as IL-12 and IFN-γ, and highly active NK cells are reportedly associated with a lower risk of cancer development [101,102,103]. In addition, *L. acidophilus* 36YL shows no apparent cytotoxicity toward normal cells, but displays high toxicity toward cancer cell lines such as HeLa and HT-29 [104]. 

Although several in vivo and in vitro studies have reported that the administration of various *Lactobacillus* strains leads to the inhibition of cancer development and progression through pro-apoptotic, antioxidant, anti-proliferative, or immunomodulatory pathways, clinical population trials and epidemiological studies are still lacking. Indeed, some issues should be carefully considered in the administration of probiotic *Lactobacillus* strains for the treatment and prevention of cancers. In particular, the potential for undesirable side effects resulting from host–bacteria interactions, individual resistance to particular strains, or variability in the benefits conferred by different *Lactobacillus* strains, all warrant further experimental investigation [80].

## 4. Oxidative Stress-Related Liver Disease

### 4.1. Oxidative Stress and Liver Disease 

Alcoholic Liver Disease (ALD): Long-term and excessive alcohol consumption is the leading cause of ALD, which includes a broad clinical-histological spectrum ranging from simple steatosis and hepatitis, to liver cirrhosis and hepatocellular carcinoma [105]. Disruption of intestinal microbiota, increased intestinal permeability, inflammatory response, liver oxidative stress, and lipid accumulation are all associated with the progression of ALD [106]. In particular, the role of oxidative stress in the occurrence and development of ALD is currently the focus of increasing research attention. Alcohol is mainly metabolized in the liver, leading to elevated ROS/RNS production, weakening antioxidant defense systems, and finally resulting in oxidative stress in the liver due to excess ROS/RNS. It should be noted that oxidative stress in the liver has been reported to play a key role in hepatocellular carcinogenesis [107].

Non-alcoholic fatty liver disease (NAFLD): NAFLD is mainly due to the accumulation of lipids in the liver, or insulin resistance caused by obesity, type 2 diabetes, and lipid metabolism disorders, among other conditions, resulting in hepatic steatosis. In addition, lipid peroxidation, oxidative stress, and pro-inflammatory cytokines are also involved, culminating in hepatocyte infiltration and necrosis. Although NAFLD has multiple clinical manifestations, most patients present with only simple steatosis [108,109].

### 4.2. The Preventive Effects of Lactobacillus

Preventive effects on ALD: *Lactobacillus* has been tested in both the treatment and prevention of ALD. Forsyth et al. found that rats fed with alcohol plus *L. rhamnosus* GG (ALC + LGG) had significantly lower severity of alcoholic steatohepatitis (ASH) than rats fed with alcohol alone, while strain LGG was also associated with significantly less alcohol-induced leakiness in the gut, oxidative stress, and inflammation in both the intestinal and liver tissues [110]. Another study comparing treatments of *Lactobacillus* strains with the traditional Chinese medicine Hu-Gan-Pian in a rat model of alcohol-induced enteric dysbiosis found that *L. rhamnosus* CCFM1107 administration could rescue dysbiosis and inhibit the characteristic increase in serum aminotransferase and endotoxin as well as triglyceride (TG) and cholesterol (CHO) levels in the serum and liver. In addition to these findings, the oxidation levels, indicated by the malondialdehyde contents, were lower, while the antioxidant enzyme (GSH/GSH-Px/SOD) levels were elevated in rats treated with *L. rhamnosus* CCFM1107 [111]. 

**Figure 3:**
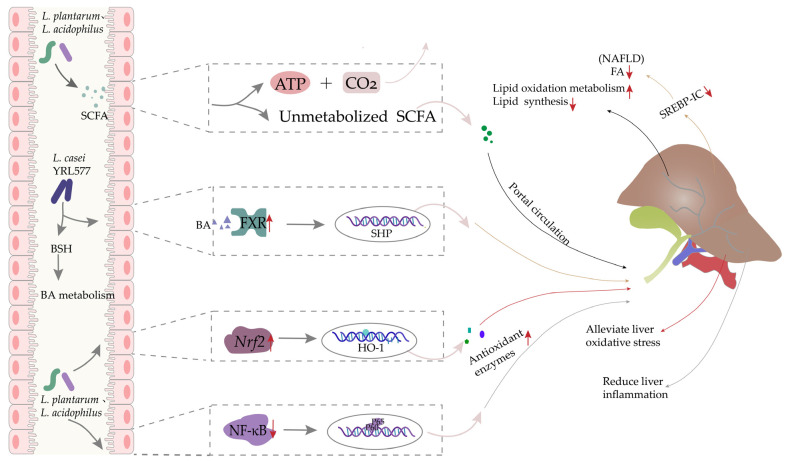
The effects of *Lactobacillus* treatment in oxidative stress-related liver disease. Some *Lactobacillus* regulate hepatic lipid metabolism and synthesis via SCFA production and the FXR/SHP/SREBP1c pathway, and some others reduce liver inflammation and oxidative stress through Nrf2 and NF-κB pathways.

In mice with alcoholic subacute liver injury, treatment with *L. plantarum* ZS62, isolated from naturally fermented yogurt, could relieve morphological abnormalities in hepatocytes and reduce markers of liver damage (e.g., AST/ALT/hyaluronidase, etc.). Furthermore, inflammation-related genes were significantly downregulated while lipid- and oxidative-metabolism genes were upregulated, implying that *L. plantarum* ZS62 could potentially function as a beneficial prophylactic supplement for people with frequent alcohol consumption [112]. In a study examining alcoholic liver injury using a zebrafish model, *L. plantarum* was shown to provide some protective effects via the transcriptional activation of the Keap-Nrf2-ARE signal pathway [113]. In work by Li and coworkers investigating the potential mechanism and effects of *Lactobacillus* in alleviating ALD symptoms in mice, treatment with a mixture of *L. plantarum* KLDS1.0344 and *L. acidophilus* KLDS1.0901 resulted in higher SCFA production by the gut microbiota. These SCFAs could potentially inhibit alcohol-induced lipid accumulation, oxidative stress, and inflammation in the liver through hepatic-intestinal circulation, most likely via activation of the AMPK, Nrf2, and TLR4/NF-κB pathways [106] (Figure 3). Collectively, these studies show that *Lactobacillus* strains could provide preventive effects against mild ALD, but long-term experiments in animal models are necessary to determine whether these strains could also alleviate end-stage ALD. 

Preventive effects of *Lactobacillus* in NAFLD: One study in rats with induced NAFLD showed that administering a *Lactobacillus*-rich mixture of probiotic strains including *L. acidophilus, L. casei,* and *L. reuteri*, could significantly reverse hepatic and blood triglyceride concentrations and blood glucose levels while suppressing markers of oxidative stress in liver tissue [114]. Zhang et al. isolated *L. casei* YRL577, which exhibited high bile acid hydrolase (BAH) activity that could regulate bile acid metabolism by increasing the transcription of fibroblast growth factor 15 (FGF15), while downregulating the host mRNA levels of Na-dependent bile acid transporter (ASBT). In addition, the YRL577 strain reduced the de novo synthesis of fatty acids (FA) via the FXR/SHP/SREBP1c pathway. These regulatory effects could possibly contribute to alleviating NAFLD in C57BL/6 mice [109] (Figure 3). Furthermore, a population-based study confirmed that dietary supplementation with probiotic strains *Bifidobacterium longum* and *L. acidophilus* could improve some anthropometric, inflammatory, and oxidative indices in patients with NAFLD [115].

Other studies have also examined the mechanisms by which *Lactobacillus* could affect NAFLD. Nrf2 is a known protective factor against liver damage, and has been implicated in the pathogenesis of chronic conditions such as NAFLD. Notably, a recent study has identified 5-methoxyindoleacetic acid, produced by the human commensal LGG, which can potently activate Nrf2 in both Drosophila liver analog and the murine liver. This activation of Nrf2 has been shown to protect against two models of oxidative liver injury, namely, acetaminophen overdose and acute ethanol toxicity [116]. Chen et al. found that treatment with *L. mali* APS1 led to reduced hepatic lipid accumulation via regulating the SIRT-1/PGC-1α/SREBP-1 pathway, and promoted hepatic antioxidant activity through the Nrf2/HO-1 pathway [117]. Another study reported that *L. plantarum* NA136 could improve NAFLD by regulating fatty acid metabolism and oxidative stress defense pathways via AMPK and Nrf2 signaling, respectively [118]. Similarly, another report suggested that *L. plantarum* NCU116 might ameliorate NAFLD by downregulating lipogenesis while promoting the upregulation of lipolysis and fatty acid oxidation-related gene expression [119]. However, the current body of literature examining the preventive or therapeutic effects of *Lactobacillus* on NAFLD remains limited. Furthermore, most of the available studies have examined mechanisms related to the regulation of lipid metabolism and the alleviation of oxidative stress, although other mechanisms are likely involved in the impacts of *Lactobacillus* on NAFLD.

*Lactobacillus* strains prevent other types of liver damage: In an animal model of carbon tetrachloride (CCl4)-induced liver fibrosis, *L. plantarum* HFY15 was found to alleviate liver injury through antioxidant, anti-inflammatory, and anti-apoptotic pathways [120]. In addition, selenium-enriched probiotics (SP) might also influence pro- and anti-apoptosis pathways, activate the SIRT1 signaling pathway, or attenuate MAPK signaling, leading to reduced liver fibrosis [121,122]. *Lactobacillus* strains were also shown to alleviate liver injuries caused by other drugs such as D-galactose (D-Gal)/LPS-induced acute liver injury (ALI) in mice or liver toxicity caused by deoxynivalenol (DON), an extremely common environmental pollutant [123,124] (Table 1).

## 5. Conclusions and Prospects

In summary, oxidative damage accompanies inflammation, cancer development, and liver injury, whereas the administration of probiotic *Lactobacillus* strains shows potential for modulating oxidative stress in cells and tissues throughout the human body, especially in the gastrointestinal tract [18]. Treatment with probiotic *Lactobacillus* supplements can also reportedly prevent or ameliorate IBD by downregulating the expression of inflammatory factors while promoting an increase in related antioxidant enzymes, possibly through the activation of various host signaling pathways. 

In cancers, it remains largely controversial as to whether targeting ROS, either by antioxidant supplements or chemical/genetic inhibition, is clinically beneficial or detrimental for cancer treatment. Several studies in animal models support the tumor suppressive effects of *Lactobacillus* strains, although it also remains uncertain whether these effects are the result of directly alleviating host ROS or due to stimulating the host antioxidant systems. Further mechanistic insights are therefore necessary to better understand the role of ROS in different types of cancer and to develop more effective, targeted probiotic anti-cancer therapies [60]. Treatment with *Lactobacillus* strains has also been shown to confer prophylactic or therapeutic effects, alleviating inflammation and oxidative damage in ALD, NAFLD, and other types of liver injury in vitro and in vivo. 

Since the large majority of the above studies are based on animal and cellular models, multi-level, multi-center population-based trials such as prospective epidemiological studies are necessary for a robust assessment of the effectiveness of probiotic treatments in IBD, cancers, and liver diseases. 

In addition, *Lactobacillus* species share conserved microbe-associated molecular patterns (MAMPs) with other Gram-positive bacteria such as cell wall polysaccharides and lipoteichoic acids, which can be recognized by the human immune system and elicit a host inflammatory response. Advances in probiotic development will thus focus on the discovery or engineering of *Lactobacillus* strains with lower MAMP-induced immunogenic effects, but with a high antioxidant capacity to maximize their therapeutic benefits in different diseases.

## Figures and Tables

**Table 1:** Summary of the cited *Lactobacillus* strains and their function in disease.

Lactobacillus Strain	Diseases Involved	Reported Functions	Reference
*L. delbrueckii* subsp. *bulgaricus* B3, *L. delbrueckii* subsp. *bulgaricus* A13	IBD	Produces EPS and antioxidant	[41]
*L. paracasei* IJH-SONE68	UC	Produces EPS	[42]
*L. plantarum* ZS62	IBD	Regulates oxidative stress and immune response	[43]
*L. casei*, *L. acidophilus*, *L. rhamnosus*	IBD	Modulates inflammatory response and oxidative stress.	[44]
*L. fermentum* ME-3	/(a healthy volunteer trial)	Antioxidant activity in healthy volunteers	[46]
Probiotic bacterial strains VSL#3	UC	Maintain remission among patients with active UC	[47]
Engineered *L. casei* BL23	CD	Candidate strain for genetic engineering	[50]
*L. delbrueckii* subsp. *Bulgaricus*, *S. thermophilus* CRL 807	Colitis	Anti-inflammatory effects	[51]
*L. casei* Shirota	Cellular (caco-2 cells) inflammatory damage and oxidative stress induced by DPPH	Alleviate oxidative stress and inflammation via Nrf2 and NF-κB pathway	[53]
*L. acidophilus* NCFM	Orthotopic colon cancers	Attenuates tumor growth and pro-apoptotic effects	[84]
*L. acidophilus*, *L. casei*	Colorectal cancer	Enhances apoptosis	[85]
LGG	Colon cancer	promotes apoptosis	[86]
LGG	HGC-27 human gastric cancer cells	Anti-proliferative effects	[87]
*L. reuteri* ATCC PTA 6475	Human chronic myeloid leukemia-derived cells	Pro-apoptotic and anti-proliferation effects	[88]
*L. casei*	Gastric cancer cells	Pro-apoptotic effects	[89]
LGG	Gastric and colonic neoplasms	Anti-proliferative effects	[90]
*L. paracasei* subsp. *paracasei* X12	HT-29 colon cancer cells	Induce cell cycle arrest at G1 (anti-proliferative)	[91]
*L. salivarius* FP25, *L. salivarius* FP35	Colon cancer cells	Anti-proliferative effects	[93]
*L. gasseri* strains	Cervical cancer cells (HeLa)	Inhibits cancer cell growth; Modulates immune response	[94]
*L. plantarum* NCU116	Colorectal cell line CT26	Regulates cancer cell proliferation and apoptosis by EPS	[95]
*L. salivarius* REN	Oral cancer	Induces apoptosis and protects against oxidative DNA damage	[97]
*L. plantarum* AS1	Colorectal cancer	Antioxidant effects	[99]
*L. acidophilus* 606	HT-29 colon cancer cells	Induces apoptosis; Antioxidant effects	[100]
*L. casei* Shirota	Normal blood mononuclear cells and splenocytes	Enhances NK cell activity	[101]
*L. casei* ssp. *casei*	Normal mice	Elicits NK cell activities.	[102]
*L. casei* Shirota	Cancer	Immunomodulatory effects	[103]
*L. acidophilus* 36YL	Human cancer cell lines (AGS, HeLa, MCF-7, and HT-29)	Cytoxicity toward cancer cells	[104]
*L. plantarum* KLDS1.0344; *L. acidophilus* KLDS1.0901	ALD	Inhibits liver lipid accumulation, oxidative stress, and inflammation. Regulates gut microbiota	[106]
*L. casei* YRL577	NAFLD	Modulates genes in intestinal bile acid pathway	[109]
LGG	ALD	Reduces oxidative stress and inflammation	[110]
*L. rhamnosus* CCFM1107	ALD	Reduces oxidative stress; Restores the intestinal microbiota	[111]
*L. plantarum* ZS62	Alcohol-induced subacute hepatic damage	Reduces inflammation; Enhances antioxidative	[112]
* L. plantarum *	ALD	Antioxidant effects via activation of Keap-Nrf2-ARE pathway	[113]
*L. acidophilus*, *L. casei*, *L. reuteri*	NAFLD	Antioxidant effects	[114]
*Bifidobacterium longum*; *L. acidophilus*	NAFLD	Mitigates oxidative stress and inflammatory response; Improves lipid profiles	[115]
LGG	NAFLD	Active Nrf2	[116]
*L. mali* APS1	NAFLD	Modulates lipid metabolism and antioxidant activity	[117]
*L. plantarum* NA136	NAFLD	Regulates the fatty acid metabolism and defends against oxidative stress via AMPK and Nrf2 pathways	[118]
*L. plantarum* NCU116	NAFLD	Regulates microbiota and lipid metabolism	[119]
*L. plantarum* HFY15	Liver fibrosis	Antioxidant; Anti-inflammatory; Anti-apoptotic effects	[120]
Se-enriched probiotics	Liver fibrosis	Attenuate hepatic oxidative stress, ER stress, and inflammation	[121]
Se-enriched probiotics	Liver fibrosis	Attenuate hepatic oxidative stress and inflammation; Induce apoptosis of hepatic stellate cells.	[122]
LGG	Deoxynivalenol exposure induces liver damage	Reduces DON toxicity	[123]
*L. plantarum* KSFY06	Acute liver injury induced by D-Gal/LPS	Anti-oxidant and anti-inflammatory activities	[124]

## Data Availability

Data is contained within the article or Supplementary Material.

## Supplementary Materials

The following supporting information can be downloaded at: https://www.mdpi.com/article/10.3390/antiox12030769/s1, Table S1: Abbreviations.
