# Supplementary material for: Recent Perspective of Lactobacillus in Reducing Oxidative Stress to Prevent Disease
Source: Antioxidants (Basel). 2023 Mar 21;12(3):769. doi: 10.3390/antiox12030769 (PMC10044891; doi:10.3390/antiox12030769)
Supplement: Supplementary file 1 [file antioxidants-12-00769-s001.zip › antioxidants-2255392-supplementary.pdf]

Supplementary table 1. Abbreviations.

Full name	Abbreviations
reactive oxygen species	ROS
reactive nitrogen species	RNS
nicotinamide adenine dinucleotide phosphate	NADPH
NADPH oxidase	NOX
myeloperoxidase	MPO
nitric oxide synthase	NOS
lipoxygenases	LOXs
cyclooxygenases	COXs
superoxide dismutase	SOD
catalase	CAT
glutathione peroxidase	GPX
glutathione reductase	GSR
glutathione S-transferase	GST
melatonin	MEL
thioredoxin	Trx
hydrogen peroxide	H_2O_2
natural killer cells	NK cells
exopolysaccharides	EPS
Inflammatory bowel disease	IBD
Crohn's disease	CD
ulcerative colitis	UC
lipid peroxidation	LPO
NAD(P)H: quinone oxidoreductase 1	NQO1
Nuclear factor E2-related factor 2	Nrf2
dextran sulfate sodium	DSS
Total Antioxidant Status	TAS
lipopolysaccharide	LPS
Nuclear Factor-Kappa B	NF-κB
Antioxidant Response Elements	AREs
8-oxo-7-hydrodeoxyguanosine	8-oxodG
extracellular signal-regulated kinase	ERK
c-Jun N-terminal kinase	JNK
p38 Mitogen-activated protein kinases	MAPK
phosphatidylinositol-3-kinase	PI3K
phosphatase and tensin homolog deleted on chromosome 10	PTEN
protein kinase B	AKT/PKB
4E-binding protein 1	4EBP1
short-chain fatty acids	SCFAs
4-nitroquioline 1-oxide	4NQO
1,2-dimethylhydrazine	DMH
Alcoholic Liver Disease	ALD

Non-alcoholic fatty liver disease	NAFLD
alcoholic steatohepatitis	ASH
bile acid hydrolase	BAH
fibroblast growth factor 15	FGF15
Na-dependent bile acid transporter	ASBT
selenium-enriched probiotics	SP
D-galactose	(D-Gal)
acute liver injury	ALI

In the order of appearance in the review
